# A Lon-Like Protease with No ATP-Powered Unfolding Activity

**DOI:** 10.1371/journal.pone.0040226

**Published:** 2012-07-06

**Authors:** Jiahn-Haur Liao, Chiao-I Kuo, Ya-Yi Huang, Yu-Ching Lin, Yen-Chen Lin, Chen-Yui Yang, Wan-Ling Wu, Wei-Hau Chang, Yen-Chywan Liaw, Li-Hua Lin, Chung-I Chang, Shih-Hsiung Wu

**Affiliations:** 1 Institute of Biological Chemistry, Academia Sinica, Taipei, Taiwan; 2 Biodiversity Research Center, Academia Sinica, Taipei, Taiwan; 3 Institute of Molecular Biology, Academia Sinica, Taipei, Taiwan; 4 Institute of Biochemical Sciences, National Taiwan University, Taipei, Taiwan; 5 Institute of Chemistry, Academia Sinica, Taipei, Taiwan; University of South Florida College of Medicine, United States of America

## Abstract

Lon proteases are a family of ATP-dependent proteases involved in protein quality control, with a unique proteolytic domain and an AAA^+^ (ATPases associated with various cellular activities) module accommodated within a single polypeptide chain. They were classified into two types as either the ubiquitous soluble LonA or membrane-inserted archaeal LonB. In addition to the energy-dependent forms, a number of medically and ecologically important groups of bacteria encode a third type of Lon-like proteins in which the conserved proteolytic domain is fused to a large N-terminal fragment lacking canonical AAA^+^ motifs. Here we showed that these Lon-like proteases formed a clade distinct from LonA and LonB. Characterization of one such Lon-like protease from *Meiothermus taiwanensis* indicated that it formed a hexameric assembly with a hollow chamber similar to LonA/B. The enzyme was devoid of ATPase activity but retained an ability to bind symmetrically six nucleotides per hexamer; accordingly, structure-based alignment suggested possible existence of a non-functional AAA-like domain. The enzyme degraded unstructured or unfolded protein and peptide substrates, but not well-folded proteins, in ATP-independent manner. These results highlight a new type of Lon proteases that may be involved in breakdown of excessive damage or unfolded proteins during stress conditions without consumption of energy.

## Introduction

Proteases play a crucial role in cellular protein quality control to degrade aberrant or unwanted proteins, which may be prone to toxic aggregate formation, into short peptides. Most of these specialized proteases are oligomeric and form an enclosed chamber, inside which the proteolytic sites are located and accessible only through a narrow axial pore [1]. In most cases, selected protein substrates are fed through the axial pore into the degradation chamber of the protease in an energy-dependent process by a ring of built-in motor subunits or domains containing conserved motifs for ATP binding and hydrolysis found in the superfamily of AAA^+^ (ATPases associated with various cellular activities) [2].

Lon proteases are key components of the protein quality-control systems in bacteria and eukaryotic organelles responsible for degrading abnormal or unfolded proteins; they are also involved in regulation of many cellular processes by selective degradation of regulatory proteins [3]. Lon was originally identified from *Escherichia coli* (hereafter EcLonA) and was the first known ATP-dependent protease [4]. Almost all Lon proteases possess two important functional domains in a single polypeptide chain: the AAA^+^ domain, which contains conserved motifs (Walker motifs) for ATP binding and hydrolysis, and the C-terminal protease domain with a conserved serine-lysine catalytic dyad in its proteolytic active site [5]. Based on the consensus sequences at the proteolytic sites in the protease domains, two subfamilies of Lon proteases, LonA and LonB, have been classified previously [6]. In general, the two subfamilies are associated with two distinct consensus sequences of the Walker motifs in the AAA^+^ domains and specific domain organization features in which an N-terminal domain is attached to the AAA^+^ modules of all LonA but not LonB members, the latters are found only in archaebacteria and possess instead additional membrane-spanning segments integrated into their AAA^+^ domains that anchors the proteins to the membrane [7]. Such proteolytic site-based classification approach, however, has left out a large group of Lon-like proteases encoded in the genomes of many Gram-negative bacteria (also in certain Gram-positive bacteria and archaea), which contain LonB-like consensus sequences at the proteolytic sites but have no transmembrane regions nor detectable AAA^+^ consensus motifs [6], [8]. Recently, the lack of ATPase activity has been confirmed for a Lon-like protease from *Thermus thermophilus* [9]; however, the biochemical properties and functional roles of these Lon-like proteases have remained largely uncharacterized.

For the present work, we performed multiple sequence comparisons of LonA, LonB, and the Lon-like proteases, which revealed additional sequence features in the protease domain unique only to the latter but not present in the ATP-dependent LonA/B members. Phylogenetic analysis based on sequence alignment results indicated that these Lon-like proteins with unique conserved insertions belong to a clade of Lon proteases distinct from the LonA and LonB groups. This group of Lon-like proteases may be assigned to a new subfamily, LonC, following previous classification scheme [6]. Here we report the biochemical and biophysical characterizations of a Lon-like protease from *M.* *taiwanensis* [10].

**Figure 1:**
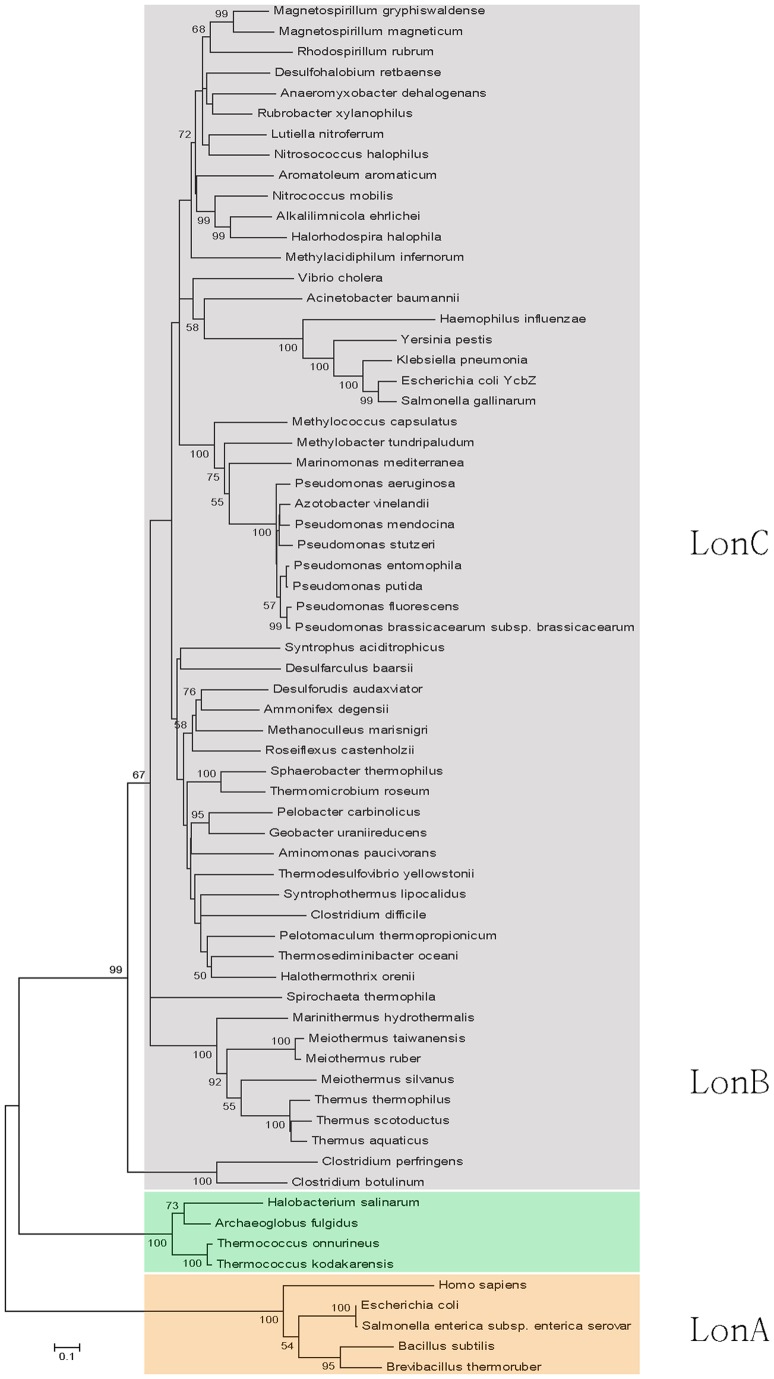
Phylogenetic tree calculated from amino acid sequence alignment of Lon-like proteases (LonC) with representative members of LonA and LonB subfamilies. Three distinct clades can be recognized through phylogenetic reconstruction. Indicated are the names of species where at least one member of the corresponding subfamily of Lon proteases is present. Note that LonA proteases are ubiquitous in all species shown; thus some bacteria, including *E. coli*, possess more than one subfamilies of Lon. In addition, multiple paralogs related to LonA have been found in certain bacteria. The Lon genes are indicated by binomial names of the species. The accession codes for the genes can be found in Table S1.

## Results and Discussion

### Sequence Alignment and Phylogenetic Analysis

We have recently sequenced the total genome of *Meiothermus taiwanensis* WR-220 and identified three Lon homologs in this indigenous thermophilic Gram-negative bacterium. While two were LonA-like, the third gene encoded a novel Lon-like protein of 719 amino acids (hereafter MtaLonC) with a conserved C-terminal Lon protease domain but containing neither LonA-like N-terminal domain, nor transmembrane regions associated only with LonB or the conserved features of AAA^+^ modules (see below). MtaLonC expressed in *M. taiwanensis* at 55°C, the organism’s growth temperature, and the expression was induced at 65°C [Fig. S1]. We conducted BLAST searches of the protein databases and identified a large number of bacterial sequences with BLAST E-values suggesting homology with MtaLonC. Most of these Lon-like proteases are found in the proteomes of Gram-negative bacteria, of which many belong to the medically important class of γ-proteobacteria, which includes the genera of *Acinetobacter, Klebsiella, Pseudomonas, Salmonella, Vibrio,* and *Yersinia*. We performed multiple sequence alignment for these Lon-like proteins, along with studied members from the LonA and LonB subfamilies, and compared the results using three different programs: MUSCLE, T-Coffee, and ClustalW2 [11]–[13]. A representative phylogenetic tree calculated from the MUSCLE alignment of Lon homologs is presented in Figure 1. It is clear that three major clades, evident in all phylogenies calculated using each of the three different alignments, are present and two of them correspond to the previously classified LonA and LonB subfamilies [6]. Using the same naming scheme, here the third group was tentatively assigned as LonC. It is noteworthy that LonB and LonC may have derived from a common ancestor, which in turn shares a common ancestor with LonA (Fig. 1, Table S1).

**Figure 2:**
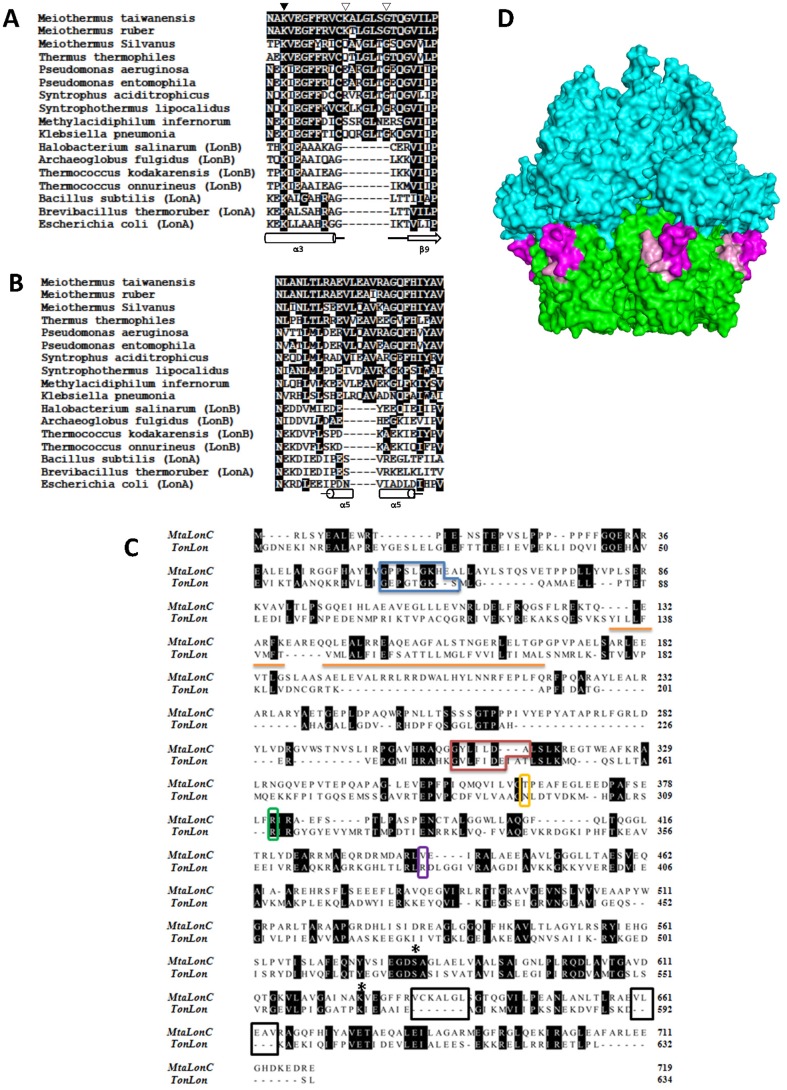
Two LonC-specific insertions. (A) Sequence alignment of selected Lon proteases (LonA and LonB members are indicated accordingly) centered on the loop between helix α3 (see text), which houses the catalytic lysine (K625 in *M. taiwanensis*; close triangle), and strand β9 in the protease domain. Open triangles mark the insertion region. (B) Alignment detail of the C-terminal region of Lon protease domain focused on helix α5. (C) Sequence alignment of MtaLonC and TonLonB. Regions corresponding to the Walker A and B motifs of TonLonB are outlined in blue and red boxes, respectively. The two LonC-specific insertions is in black box. The MtaLonC residues corresponding to the sensor-1, sensor-2, and Arg finger of TonLonB are in boxes of yellow, purple, and green colors, respectively, for comparison (see text). The transmembrane regions of TonLon are underlined. Conserved residues are highlighted in black blocks. Catalytic dyad residues are marked with asterisks. (D) Locations of the two LonC-specific insertions, in the α3-β9 loop (colored in magenta) and helix α5 (pink) of the Lon protease domain (green), mapped onto a surface representation of hexameric TonLon (PDB code 3K1J).

Alignment of the conserved Lon protease domains from all three subfamilies revealed two insertions unique to the LonC group (K634–G640 and V660–V664 of MtaLonC)(Fig. 2A, B and C). According to the crystal structure of the protease domain of EcLonA, one insertion has a consensus sequence of CX_3_GLX(G/E) and is located in the loop connecting the helix α3 and strand β9, two structural elements conserved in all known structures of isolated Lon protease domains [14]–[18]. The other insertion with a consensus sequence of φX_2_Aφ (φ = hydrophobic amino acid) is within helix α5, which adapts an alternative loop conformation in two structures [14], [18]. Based on the recent structure of a hexameric LonB (TonLonB; PDB code 3K1J) [19], all structural elements (α3, α5, and β9) are on the outer surface of the degradation chamber (Fig. 2D). Interestingly, they are located at the contact interface between the α subdomain of the ATPase module and the protease domain; hence the LonC-specific insertions may affect the communication between the two functional units.

**Figure 3:**
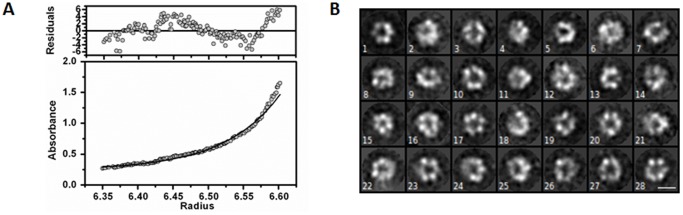
Hexameric assembly of MtaLonC. (A) Molecular weight analysis of MtaLonC by analytical ultracentrifugation. Representative sedimentation equilibrium distribution of Mt-LonC suggested an average molecular weight of 462,476 Da, corresponding to hexamers in solution. (B) Representative class averages of negatively stained MtaLonC particles. Images such as 1, 15, 20, and 23 show the predominant top view of the hexameric complex. Images 3, 5, 7, and 27 may represent various side views of the assembly. The scale bar is 10 nm.

### Hexameric Assembly without Mg^2+^ or Nucleotides

Full-length MtaLonC was cloned and expressed in *E. coli* as a C-terminal His-tagged protein, which appeared to be well-folded (Fig. S2). The protein had a tendency to form aggregates eluted as a sharp peak at the void volume when concentrated but appeared as a single hexameric species at 1 mg/mL in pH 5–8, with a molecular mass of 420–480 kDa as judging by size-exclusion chromatography. Formation of MtaLonC hexamers was not dependent on Mg^2+^ or ATP, a property different from EcLonA [20]. The hexameric assembly was also confirmed by analytical ultracentrifugation, from which an apparent molecular weight (Mw.) was estimated of 460 kDa, corresponding to a hexamer (Fig. 3A and Fig. S3; monomer Mw. = 79 kDa). To examine the oligomeric assembly in further detail, we performed electron microscopic (EM) analysis. Electron micrographs of the hexamer and void-volume fractions from Superose 6 all showed a uniform size of negatively stained particles with two general forms (Fig. 3B) and no larger particles with a higher ordered structure could be observed in the void-volume fraction. Most of the particles with a likely *en face* orientation appeared as symmetric hexamers (for example, images 1, 15, 20, and 23), with a dimension of 13 nm comparable to that of hexameric TonLonB (120 Å). Also observed were egg-shape particles with central dark region implying a hollow chamber (images 5, 10, 26 and 27). In some cases, tunnel-like feature could be identified as well, which together with the hollow chamber were also found in the hexameric structure of TonLonB [19].

**Figure 4:**
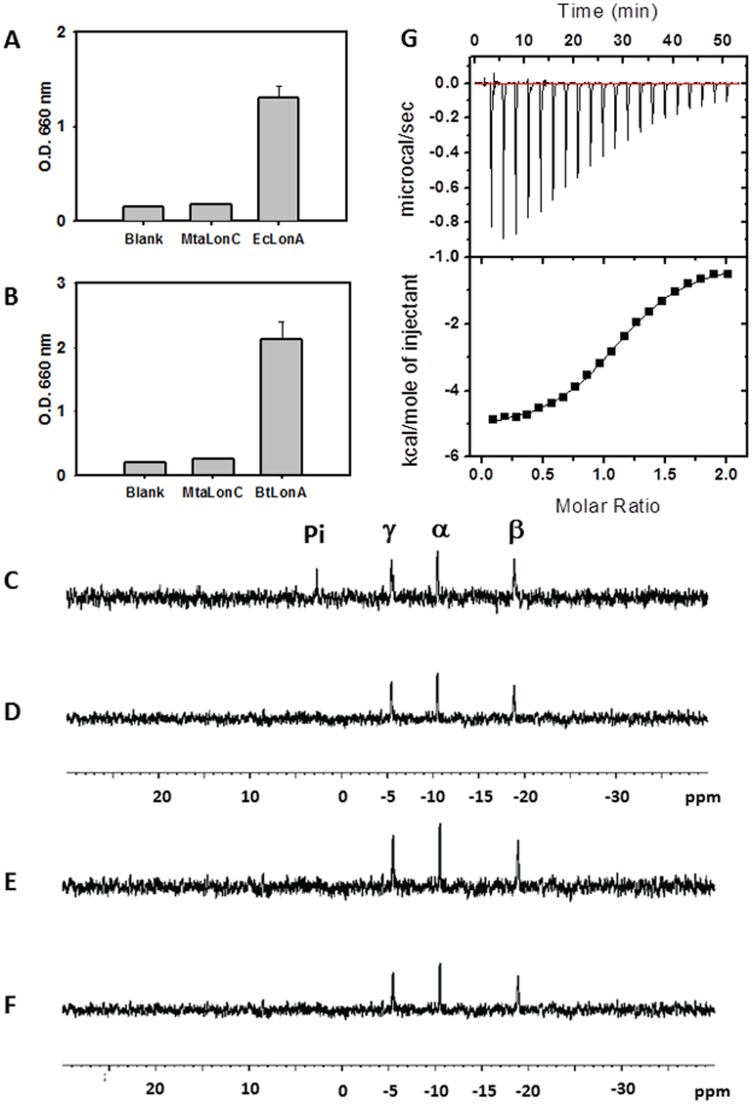
ATPase and ATP-binding activities of MtaLonC. ATPase activity of MtaLonC were assayed at 37°C with EcLonA as a positive control (A) and at 55°C, the growth temperature of *M. taiwanensis*, using BtLonA as a positive control (B). Blank, no enzyme added. (C, D) ^31^P-NMR spectra of 10 mM ATP in the presence of EcLonA (2 µg) at 37°C for 5 days (C) and 1 hour (D). (E, F) ^31^P-NMR spectra of 10 mM ATP in the presence of MtaLonC (2 µg) at 37°C for 5 days (E) and 1 hour (F). The positions of chemical shifts for the phosphorus atoms of ATP and of inorganic phosphate (Pi) are indicated. Four peaks are shown in figure C indicates that EcLonA possesses ATPase activity whereas MtaLonC does not. (G) Binding isotherms of MtaLonC titrated with ATPγS showing a binding ratio (n) of 1.15 (fitted by one-site function). The association constant (K), ΔH, and ΔS was 1.28×10^5^ (M^−1^), −5283 (cal/mol), and 5.65 (cal/mol/deg), respectively. ΔG was −6.97 (kcal/mol) indicating a spontaneous binding.

### Lack of ATPase Activity

One of the main sequence features of the LonC proteases is their lack of canonical AAA^+^ motifs. Previously, a similar Lon-like protein TTC1975 from *Thermus thermophilus*, which belongs to the LonC subfamily (Fig. 1), was shown to have no ATPase activity and its proteolytic activity was not stimulated by the addition of ATP [9]. Similar to TTC1975, we found no ATPase activity associated with MtaLonC at either 37°C or 55°C, the optimal growth temperature for the thermophilic bacteria, using a colorimetric assay [21], [22]; by contrast, the activity was detected for EcLonA, as well as a LonA protease from the thermophilic *Brevibacillus thermoruber* (BtLonA)(Fig. 4A and B). The ATPase activity was also tested by monitoring release of inorganic phosphate (Pi) from ATP hydrolysis by the enzyme by ^31^P-NMR at 37°C (Fig. 4 C–F) [23]. As shown, no peak for P_i_ was detected even after 5 days of incubation of ATP with MtaLonC (Fig. 4D), during which the protein appeared to be intact as judging by SDS-PAGE and gel filtration analyses. On the contrary, incubation of an excess amount of ATP with EcLonA resulted in hydrolysis of ATP and the appearance of a peak for P_i_ (Fig. 4F). This result confirmed that MtaLonC has no ATPase activity.

**Figure 5:**
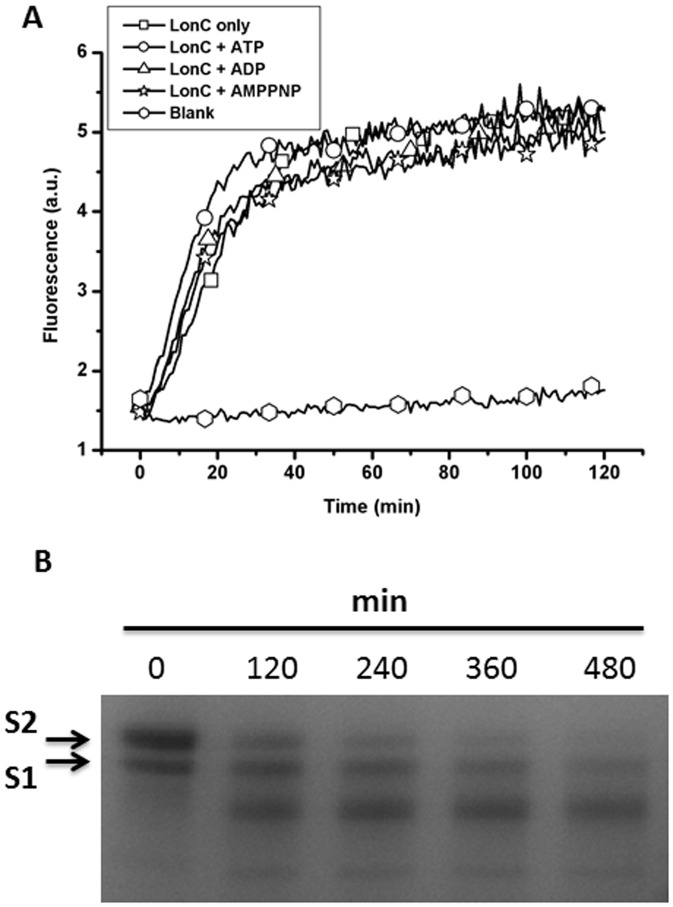
MtaLonC degrades unstructured polypeptide. (A) Degradation of F-β20-Q in the absence or presence of ATP, ADP or AMPPNP as revealed by increased fluorescence upon cleavage. (B) Degradation of α_S2_-Casein by MtaLonC at 55°C was assayed by SDS-PAGE. The sample also contained minor amount of α_S1_-Casein.

**Figure 6:**
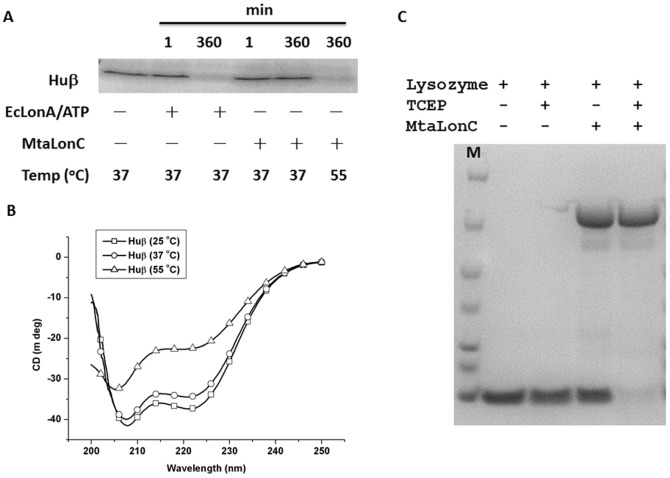
MtaLonC degrades unfolded protein substrates. (A) Degradation of Huβ by MtaLonC or EcLonA. The reactions were carried out at 37 or 55°C, with or without addition of ATP as indicated in each panel, and was analysed by SDS-PAGE. Left lanes were reaction mixtures without enzyme incubated for 360 min. (B) CD spectra of of Huβ at various temperatures. (C) Degradation of lysozyme by MtaLonC at 55°C.

**Figure 7:**
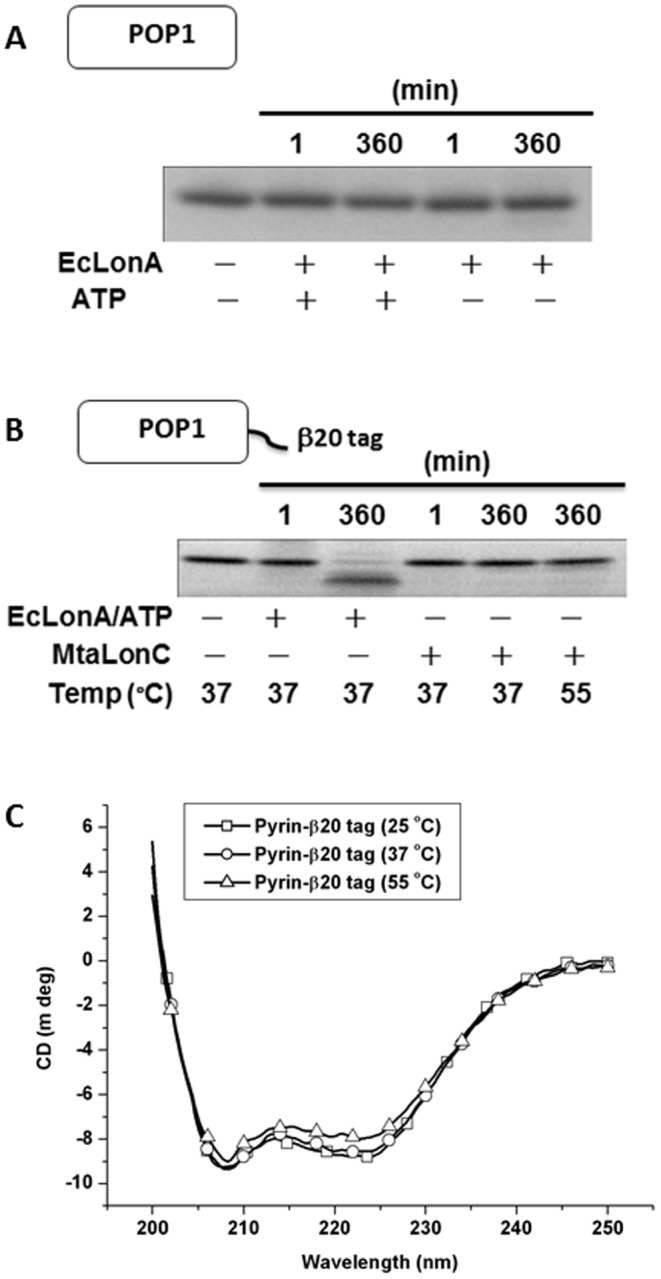
MtaLonC cannot degrade well-folded protein. (A) Degradation of POP1 by EcLonA at 37°C with or without ATP. (B) Degradation of POP1-β8 by MtaLonC or EcLonA. The reactions were carried out at 37 or 55°C, with or without addition of ATP as indicated in each panel, and was analysed by SDS-PAGE. Left lanes were reaction mixtures without enzyme incubated for 360 min. (C) CD spectra of of POP1-β8 at various temperatures.

Despite the lack of ATPase activity, we found that MtaLonC still retains an ability to bind nucleotide. Isothermal titration calorimetry (ITC) was used to analyze the binding isotherms of ATPγS and MtaLonC (Fig. 4G). The mixture of ATPγS and MtaLonC yielded exothermic titration curves indicative of binding to the nucleotide. The titration isotherm curve of the complexes was fitted with a one-site model and gave a binding stoichiometry of n = 1.15, suggesting an equal binding of 6 nucleotides per hexamer, with a K_d_ of 7.8 µM. By contrast, TonLonB exhibited a binding of 3 ATPγS per hexamer with a higher affinity (2 µM) [19]. In fact, a maximum occupancy of four nucleotides to the six available ATP/ADP binding sites in the hexameric ring of AAA^+^ modules were observed from solution studies [24]–[26], as well as in some crystal structures [19], [27], [28]. As proposed previously, such unequal binding of nucleotides may be necessary for the ATPase cycle to drive conformational changes in the hexameric AAA^+^ ring to generate mechanical power for substrate unfolding and translocation [1]. The fact that MtaLonC has no ATPase activity but binds six nucleotides in hexameric assembly points out a possibility that it may contain six degenerate AAA-like modules, which may have not lost nucleotide-binding ability but cannot generate power strokes to unfold protein substrates as canonical AAA^+^ proteases do. A strong support to this hypothesis comes from sequence alignment of TonLonB and MtaLonC, from which we identified a Walker A-like motif near the N-terminus (GPPSLGKHE; conserved residue underlined) and a Walker B-like motif (“Walker B”: GYLILDA) in the middle portion of the sequence where the conserved Glu (preceding the conserved aspartate) essential for ATP hydrolysis is missing and replaced with a leucine. The conserved AAA^+^ residues, sensor-1 and sensor-2, are replaced by non-conserved residues; and the arginine finger is disrupted by a short insertion in MtaLonC (Fig. 2C). Missing all these important AAA+ residues required for sensing and performing ATP hydrolysis in MtaLonC is consistent with its lack of ATPase activity. A structural analysis is needed to validate this hypothesis.

**Figure 8:**
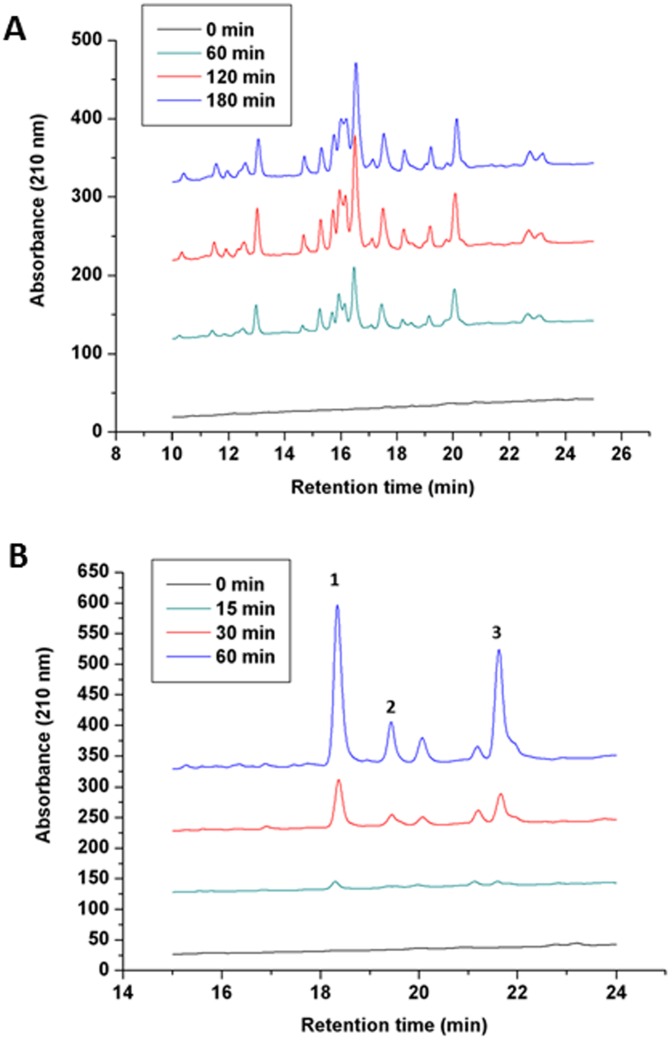
Processive degradation of MtaLonC. HPLC profiles of the degraded products from *E. coli* Huβ (A) and α_S2_-casein (B) after indicated incubation times. The cutting sites of MtaLonC in *E. coli* Huβ analyzed by mass spectrometry are shown in supplementary data (Fig.S6). Molecular species of the major peaks of casein digestion were confirmed by mass spectrometry. Peak 1: AMKPWIQPK; peak 2: TKVIPYVR; peak 3: FALPQYLK.

### Selective Degradation of Unfolded Peptide or Protein Substrates in ATP- and Divalent Cation-independent Manner

The peptidase/protease activity of EcLonA is known to rely strictly on Mg^2+^ (or other divalent cations) and ATP [29]. We found that in the absence of both, MtaLonC readily cleaved F-β20-Q, a fluorogenic peptide derived from an internal segment of β-galactosidase (QLRSLNGEWRFAWFPAPEAV), which contains all the elements required for recognition and degradation by EcLonA in the presence of ATP [30]. Using the β20 peptide, we found that MtaLonC had an optimal peptidase activity between 52 and 65°C, at pH 7.0 (Fig. S4). As shown in Fig. 5A, MtaLonC cleaved β20 at similar rates without or with added ATP, ADP, or nonhydrolyzable AMPPNP. The *K*_M_ for the ATP-independent reaction by MtaLonC was 14 µM, three-fold higher than EcLonA (4.6 µM), whereas the *V*_max_ was 50-fold lower than EcLonA (0.2 vs. 10 µM/min), the latter however had no cleavage activity at all towards β20 in the absence of ATP ([30] and data not shown). Mass spectrometry analysis of digested β20 products revealed a major cleavage site between the residues F11 and A12. The lower cleavage rate of β20 indicated that this peptide is not as good substrate to MtaLonC as to EcLonA, which may reflect their non-homologous N-terminal domains mediating substrate recognition/entry to the proteolytic chamber. Nevertheless, MtaLonC still recognizes β20 as a degron. The fact that MtaLonC was able to cleave β20 at the position where the P1 (F11) and P3 (W9) residues are hydrophobic, a result consistent with previous cleavage analysis of ribosomal S2 protein by EcLonA [31], suggests a conserved Lon proteolytic domain present in these two types of Lon. We also examined the proteolytic activity of MtaLonC using four substrates with different thermostabilities. Bovine α-caseins comprise α_S1_-casein and α_S2_-casein; both are unfolded phosphoproteins without any secondary structure. We found that MtaLonC preferably degraded α_S2_-casein but did so less well for α_S1_-casein in the absence of ATP and Mg^2+^ (Fig. 5B). Interestingly, MtaLonC did not completely degrade caseins into small peptides; a peptide product with an intermediate size was in fact accumulating in the course of digestion (Fig. 5B). α_S2_-Casein is distinguished from α_S1_-casein by the presence of clusters of negative and positive amino acids at the N- and C-termini, respectively. Intriguingly, MtaLonC could not degrade dephosphorylated α-caseins; by contrast, EcLonA can efficiently degrade all forms/isoforms of α-casein (Fig. S5) [32]. These results indicated that MtaLonC might possess a unique axial pore, which mediates substrate recognition distinct from that of EcLonA. *E. coli* Huβ has been shown to be a substrate of EcLonA (Fig. 6A) [33]. CD spectra of Huβ indicated that the protein was folded at both 25 and 37°C, but exhibited little secondary structural feature at 55°C (Fig. 6B). MtaLonC degraded the protein at 55°C, where Huβ was likely unfolded. By contrast, folded Huβ was degraded only by EcLonA but not by MtaLonC at 37°C (Fig. 6A). In a related experiment, we found that MtaLonC could not degrade hen lysozyme, which contains four internal disulfide bonds, at 55°C. However, unfolded lysozyme, denatured by TCEP treatment, was completely degraded by MtaLonC (Fig. 6C). These results strongly suggested that MtaLonC could only degrade unfolded protein substrates. As a further test, we engineered a construct, designated as POP1-β8, in which the hydrophobic core of β20 (WRFAWFPA) was cloned to the C-terminus of pyrin-only protein 1 (POP1), a stable 12-kDa α-helical protein. Although only the octapeptide core of the β20 degron was used in the fusion construct, the resulting C-terminal sequence is highly similar to β20. A similar heptapeptide core peptide of β20 has been demonstrated to inhibit degradation of F-β20-Q by EcLonA [30]. While native POP1 was not recognized and degraded by EcLonA (Fig. 7A), the enzyme degraded POP1-β8 into shorter species (Fig. 7B). On the contrary, MtaLonC could not degrade POP1-β8, which exhibited thermal stability from 25 to 55°C (Fig. 7C). Overall, these results indicated that MtaLonC has no unfolding power and thus cannot degrade folded proteins.

### Processive Protein Degradation by MtaLonC

Canonical ATP-dependent Lon proteases are known to degrade protein substrates in processive manner [31], [34]. To find out whether MtaLonC carried out degradation process in similar fashion, purified *E. coli* Huβ and α_S2_-casein were used as substrates and analyzed by reverse-phase HPLC and mass spectrometry (Fig. 8; Fig. S6). Similar to previous findings, the HPLC profiles of degradation products showed time-dependent increase of most product peptide peaks, which persisted throughout the incubation period [31], [34]. These results suggested that processivity is an inherent feature of Lon proteolytic domain and is not dependent on ATP hydrolysis.

### Conclusion

In this work, we presented biophysical and biochemical characterizations of MtaLonC, which belongs to a novel class of ATP-independent Lon-like proteases. As revealed by EM, MtaLonC likely forms a hexameric assembly with a hollow chamber. Although devoid of ATPase activity, based on sequence alignment and an ability to bind six nucleotides we propose that MtaLonC hexamer may contain a layer of six AAA-like domains and thus may exhibit an overall three-dimensional architecture similar to the recent crystal structure for the non-membrane portion of TonLonB [19]. However, in contrast to the obstructed hydrophobic pore (<5 Å) in TonLonB, MtaLonC may possess a distinct open pore wide enough to allow only unfolded peptide or protein substrates to pass through, but disallow well-folded proteins to access the degradation chamber. The energy-independent degradation of unfolded substrates by MtaLonC reported here is reminiscent of the proposed role of 20S proteasome core particle (CP) to degrade unfolded oxidized proteins in an ATP-independent and ubiquitin-independent pathway [35]. The outer rings of CP form a 13-Å entrance known as the α-annulus, through which all substrates must pass to enter the proteasome. In this regard, MtaLonC may have a similar open pore. Currently, the functional roles of the Lon-like proteases are unknown. As the energy consumption for degrading polypeptide substrates by EcLonA may be substantial [30], [36], it is tempting to speculate that the ATP-independent Lon-like proteases may offer fuel-free housekeeping solution for eliminating excessive unfolded or damaged proteins induced by various stress and oxidative conditions. However, as MtaLonC can degrade the specific α-casein isoform, they may also have regulatory roles by degrading specific short-live substrates that cannot be recognized by canonical Lon proteases. Insight into this new type of Lon-like proteases awaits identification of the specific substrates.

## Materials and Methods

### Molecular Cloning and protein purification

The thermophilic bacterium *M. taiwanensis* WR-220 was previously isolated from a hot spring in Wu-rai, Taipei County, Taiwan [10]. *M. taiwanensis* WR-220 was grown in medium containing 0.3% peptone, 0.1% yeast extract, 0.1% glutamic acid, and Castenholz salts, at pH 7.8 and 55°C. The complete genome of *M. taiwanensis* was sequenced by Genomics BioSci & Tech (Taipei, Taiwan), and the nucleotide sequence with similarities to the gene encoding the Lon protease was annotated and cloned into expression vector pET21a. The forward primer contained an ATG initiation codon and an NdeI restriction site. The reverse primer complementarily overlapped the 3′-end of the coding region and contained a HindIII site. After double-digestion with NdeI and HindIII, the product was purified and ligated into the *Nde*I/*Hin*dIII sites of the isopropyl β-d-thiogalactoside (IPTG)-inducible *E. coli* expression vector pET21a (+) (Novogen, USA). *E.* *coli* strain BL21(DE3) (Novogen, USA) was transformed with the recombinant plasmids. The nucleotide sequence of the gene encoding the Lon protease homolog MtaLonC was deposited in GenBank (accession number: GQ423488). POP1 with the FAWFP core octapeptide (WRFAWFPA) within β20 appended at the C-terminus (POP1-β20) was created by a PCR-based mutagenesis protocol [37] and the construct was verified by DNA sequencing. Proteins were expressed in BL21(DE3) cells by induction with IPTG at 20–25°C for overnight and purified from cleared cell lysates to homogeneity by sequential chromatography on Ni-Sepharose 6 fast flow, Mono Q 5/50 GL, and Superose 6 10/300 GL columns (GE Healthcare). Protein concentration was quantified with Bradford Reagent (Bio-Rad). Recombinant EcLonA and BtLonA (see main text) was expressed and purified as described previously [17], [37].

### Phylogenetic Analysis of MtaLonC

Protein sequence of MtaLonC was submitted to the BLAST network service of the Swiss Institute of Bioinformatics (SIB). Selected sequences that are highly similar to *M. taiwanensis*, together with five and four sequences from the LonA and LonB subfamilies, respectively, were downloaded for phylogenetic analysis. Sequences were aligned using the program MUSCLE [38] with default parameters. The aligned sequences were imported to MEGA 5.03 [39] for further analysis. Relationships among groups of proteases were inferred through the neighbour-joining method and the Jones-Taylor-Thornton (JTT) model implemented in MEGA. Rates among sites were uniform and gaps and missing data were completely deleted. The tree reliability was evaluated by performing 1,000 replicates of bootstrap re-sampling.

### Electron Microscopy

Diluted MtaLonC solution (∼ 50 µg/ml) was negatively stained by 2% uranyl acetate (UA). A FEI Tecnai G2 Spirit Twin transmission electron microscope operating at 120 kV was employed to serve the EM data collection. The specimen was imaged on a Gatan 1k×1k CCD at a magnification of 30000x, conferring a pixel size of 5.6 Å. The corresponding defocus values were in the range of −2.0 ∼ −2.3 µm (estimated by CTFFIND3 [40]).

### Analytical Ultracentrifugation Analysis

Sedimentation velocity was performed at 20,000 rpm with standard double-sector centerpieces at 20°C in a Beckman XL-A analytical ultracentrifuge (Beckman Instruments, Fullerton, Calif., USA) with an An-60 Ti rotor. The UV absorption (280 nm) of the cells was scanned every 4 min for 250 scans. The data were analysed with the software SEDFIT [41]. The protein sample (7 µM) was in the buffer containing 20 mM sodium citrate, 100 mM NaCl, 1 mM DTT, pH 5.5 with a concentration of 7.8 µM. All samples were visually checked for clarity after ultracentrifugation; no precipitation was observed. Sedimentation equilibrium experiment was performed using an Optima XL-I analytical ultracentrifuge (Beckman Instruments, Palo Alto, CA, USA) in an An-60 Ti rotor equipped with a standard six-channel cell. The protein sample was in the buffer containing 20 mM sodium citrate (pH 5.5), 100 mM NaCl, 1 mM DTT, with a concentration of 7.8 µM. The protein samples were rotated between speeds 4,000, 5,000 and 6,000 r.p.m. at 20°C until equilibrium was attained. Absorbance was monitored at 280 nm. The apparent molecular weight (MWapp) was obtained by global fitting of multiple scans acquired at 4,000 r.p.m. using the sedimentation analysis software supplied by the manufacturer.

### ATPase Activity Assay

The ATPase activity of MtaLonC was measured as described previously [21], [22]. The reaction mixtures contained 50 mM Tris-HCl (pH 8.0), 10 mM MgCl_2_, 1.0 mM ATP, and 5 µg MtaLonC in a total volume of 100 µl. Solutions were incubated at 37 or 50°C for 40 min, and the color of the reaction was developed by adding 800 µl malachite/molybdate solution. The reactions were terminated by adding 100 µl 34% sodium citrate. The absorbance of the final reaction was determined at 660 nm on a Beckman Coulter DU730 UV/Vis spectrophotometer.

### ^31^P-NMR

30 µM of EcLonA in buffer containing 50 mM Tris-HCl (pH 8.0), 10 mM MgCl_2_, 10 mM ATP and 30 µM of MtaLonC in buffer containing 20 mM HEPES (pH 7.5), 100 mM NaCl, 10 mM ATP were incubated at 37°C for 5 days. NMR studies were carried out on a Bruker AC 400 spectrometer.

### Isothermal Titration Calorimetry (ITC)

ITC measurements were conducted on an ITC200 Microcalorimeter (GE Healthcare, USA). The heat produced by the complex formation while 1 mM ATPγS was mixed with 0.1 mM MtaLonC at 25°C in the buffer containing 20 mM sodium citrate pH 5.5, 300 mM NaCl, 10 mM MgCl_2_ was analyzed using Origin software (Edition 7.0, Microcal Inc.).

### Peptidase Activity Assay

The fluorescence peptide assays were performed with the F-β20-Q peptide {ortho-aminobenzoic acid (Abz)- QLRSLNGEWRFAWFPAPEAV[Tyr(3-NO_2_)]A}, flanked by a pair of fluorophore (Abz) and quencher (nitrotyrosine) [30]. The reaction mixtures contained β20 peptide (5 µM), 2 mM HEPES, 10 mM NaCl, pH 7.5 with or without MtaLonC (0.4 µM) were used as positive and negative control. The samples in the buffer containing β20 peptide (5 µM), 2 mM HEPES, 10 mM NaCl, 10 mM MgCl_2_, pH 7.5 with 1 mM ATP, ADP, or AMPPNP were used as experimental groups. The reactions were carried out at 55°C. The fluorescence spectra were recorded with a Jobin Yvon Fluorolog-3 fluorescence spectrophotometer. Excitation was at 320 nm, and emission was measured at 420 nm.

### Protease Activity Assay

α_S2_-Casein was purified from α-casein (Sigma, USA) on a MonoS HR5/5 column (GE Healthcare, USA). Purified α_S2_-casein (0.5 µM), which also contained a minor amount of isoform S1, was incubated with MtaLonC (1.5 µM) in the buffer containing 0.1 mM NaCl, 20 mM HEPES pH7.5 at 60°C. POP1-β20 (0.25 mg/ml) or *E. coli* Huβ (0.2 mg/ml) were incubated with EcLonA (0.2 mg/ml) in 50 mM Tris-HCl (pH 8.0), 10 mM MgCl_2_, 10 mM ATP, and incubated with MtaLonC (0.2 mg/ml) in 0.1 mM NaCl, 20 mM HEPES pH 7.5. POP1 was incubated with *E. coli* Lon (0.16 mg/ml) in 50 mM Tris-HCl (pH 8.0), 10 mM MgCl_2_, with or without 10 mM ATP. Samples were analyzed by SDS-PAGE. Hen lysozyme (7 µg) was incubated with or without MtaLonC (5 µg) in the presence or absence of TCEP (1 mM Tris(2-carboxyethyl)phosphine) in 50 mM Tris-HCl (pH 8.0), 10 mM Na_2_HPO_4_ (20 µl) at 55°C for 3 hours and analyzed by SDS-PAGE.

### Secondary Structure Analysis

Far-UV CD spectra were recorded with JASCO J-715 spectropolarimeter. α_S2_-Casein was in the buffer containing 0.1 mM NaCl, 20 mM HEPES pH 7.5, whereas MtaLonC was in the buffer containing 10 mM Na_2_HPO_4_, 2 mM KH_2_PO_4_, 3 mM KF and 70 mM NaF, pH 7.4. The POP1-β20 (0.5 mg/ml) was in 50 mM Tris-HCl (pH 8.0), 10 mM MgCl_2_ and measured at different temperature. *E. coli* Huβ (4.0 mg/ml) was in 0.1 mM NaCl, 20 mM HEPES pH 7.5 and measured at different temperature. The far-UV CD spectra were the mean of three accumulations with a 0.1-cm light path.

### Western Blotting

*M. taiwanensis* was grown in medium containing 0.3% peptone, 0.1% yeast extract, 0.1% glutamic acid, and Castenholz salts, pH 7.8, at 55°C and 65°C. Bacteria cells were lysed in B-PER bacterial protein extraction reagent (PIERCE, USA). Bacteria lysates (100 µg) were separated by SDS-PAGE and electrotransferred to a PVDF membrane. The membranes were incubated at room temperature for 1 h in blocking solution (5% nonfat milk in phosphate-buffered saline (PBS) containing 0.1% Tween 20) and then at room temperature for 2 h with anti-MtaLonC antibody (1∶40000). After three washes in PBS with 0.1% Tween 20, the membrane was incubated with an HRP-conjugated secondary antibody (1∶50000). The membrane was developed using an enhanced chemiluminescence Western blot detection system.

### Analysis of Protein Degradation by Reverse-phase Chromatography

Bovine casein (200 µg) was incubated with MtaLonC (15 µg) in the buffer (60 µl) containing 50 mM Tris HCl, 10 mM Na_2_HPO_4_, pH 8.0 for 0, 15, 30, and 60 min. After incubation, equal volume (60 µl) of 7.4 M guanidine hydrochloride was added to stop enzyme reactions. The samples were analyzed by reverse phase HPLC (C_18_ column) with line gradient (0 to 100% acetonitile, with 0.05% trifluoroacetic acid) for 60 min. The sample of each peak was collected and analyzed by Synapt G2 HD mass spectrometer. The MS/MS data was analyzed by Mascot search.

## Supporting Information

Figure S1 **Expression of MtaLonC.** Western blotting of MtaLonC showed that more MtaLonC was expressed at 65°C. Lane 1: *Meiothermus taiwanensis* growth at 55°C for one day. Lane 2: *Meiothermus taiwanensis* growth at 65°C for one day. All samples were loaded at 100 µg of protein per lane. Arrow indicated the signal of MtaLonC.
(TIF)

Figure S2 **SDS-PAGE and CD spectra of MtaLonC.** (A) Coomassie Brilliant Blue-stained SDS polyacrylamide gel of purified MtaLonC (about 3 µg). The masses of markers are 116.0, 66.2, 45.0, 35.0, 25.0, 18.4, and 14.4 kDa from top to bottom of the gel. (B) Far-UV CD spectrum of MtaLonC. (C) Near-UV CD spectrum of MtaLonC.
(TIF)

Figure S3 **Sedimentation velocity analysis of MtaLonC.** Sedimentation coefficient distribution of MtaLonC was between 14.1S and 18.3S with a peak at 15.7S.
(TIF)

Figure S4 **Temperature- and pH-dependent peptidase activity of MtaLonC.** (A) Peptidase activity of MtaLonC at various temperatures showing an optimal temperature between 52 and 65°C. (B) Peptidase activity of MtaLonC under various pH. All fluorescence values were determined after subtracting that of the negative control.
(TIF)

Figure S5 **MtaLonC cannot degrade dephosphorylated casein.** Protease activity of MtaLonC (50 µg) against dephosphorylated FITC-casein was assayed in the absence or presence of ATP. The fluorescence was measured as described previously (*J* *Biol Chem* 279 (2004): 34903–34912; *Eur J Biochem* 271 (2004): 834–844). Emission was recorded at 525 nm. (A) Cleavage of dephosphorylated FITC-casein at 37°C. EcLonA (4 µg) was used as a positive control. (B) Degradation of the substrate at 55°C. BtLonA (4 µg) was used as a positive control. Blank, no added enzyme.
(TIF)

Figure S6 **The cutting site of MtaLonC in** ***E. coli*** **Huβ.** *E.* *coli* Huβ were degraded by MtaLonC in the buffer containing 50 mM Tris HCl, 10 mM Na_2_HPO_4_, pH 8.0 for 17 hours and analyzed by mass spectrometry.
(TIF)

Table S1 **The accession codes for the genes in phylogenetic tree.**
(DOCX)
