# Supplementary figures and images for: A Lon-Like Protease with No ATP-Powered Unfolding Activity
Source: PLoS One. 2012 Jul 6;7(7):e40226. doi: 10.1371/journal.pone.0040226 (PMC3391209; doi:10.1371/journal.pone.0040226)

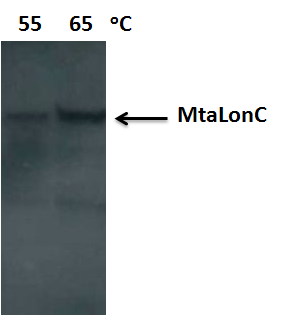

Supplement: Figure S1 — Expression of MtaLonC. Western blotting of MtaLonC showed that more MtaLonC was expressed at 65°C. Lane 1: Meiothermus taiwanensis growth at 55°C for one day. Lane 2: Meiothermus taiwanensis growth at 65°C for one day. All samples were loaded at 100 µg of protein per lane. Arrow indicated the signal of MtaLonC. (TIF) [file pone.0040226.s001.tif]

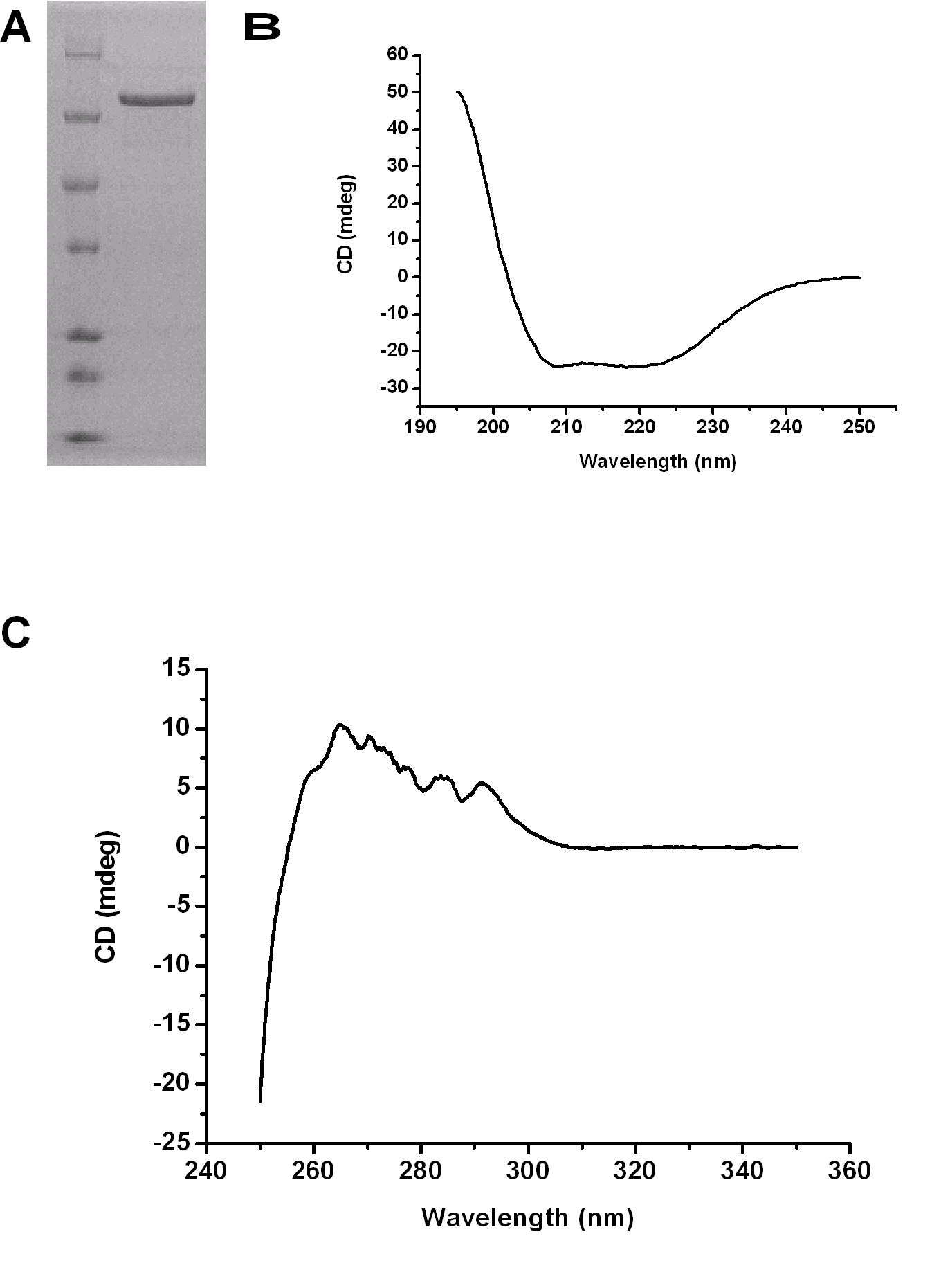

Supplement: Figure S2 — SDS-PAGE and CD spectra of MtaLonC. (A) Coomassie Brilliant Blue-stained SDS polyacrylamide gel of purified MtaLonC (about 3 µg). The masses of markers are 116.0, 66.2, 45.0, 35.0, 25.0, 18.4, and 14.4 kDa from top to bottom of the gel. (B) Far-UV CD spectrum of MtaLonC. (C) Near-UV CD spectrum of MtaLonC. (TIF) [file pone.0040226.s002.tif]

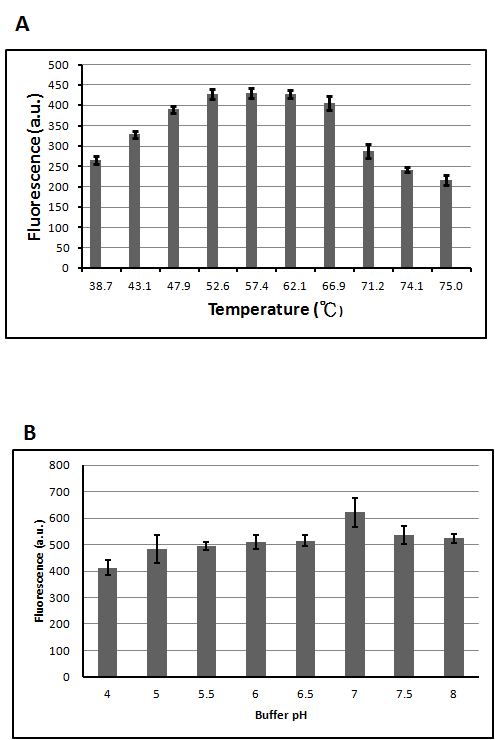

Supplement: Figure S4 — Temperature- and pH-dependent peptidase activity of MtaLonC. (A) Peptidase activity of MtaLonC at various temperatures showing an optimal temperature between 52 and 65°C. (B) Peptidase activity of MtaLonC under various pH. All fluorescence values were determined after subtracting that of the negative control. (TIF) [file pone.0040226.s004.tif]

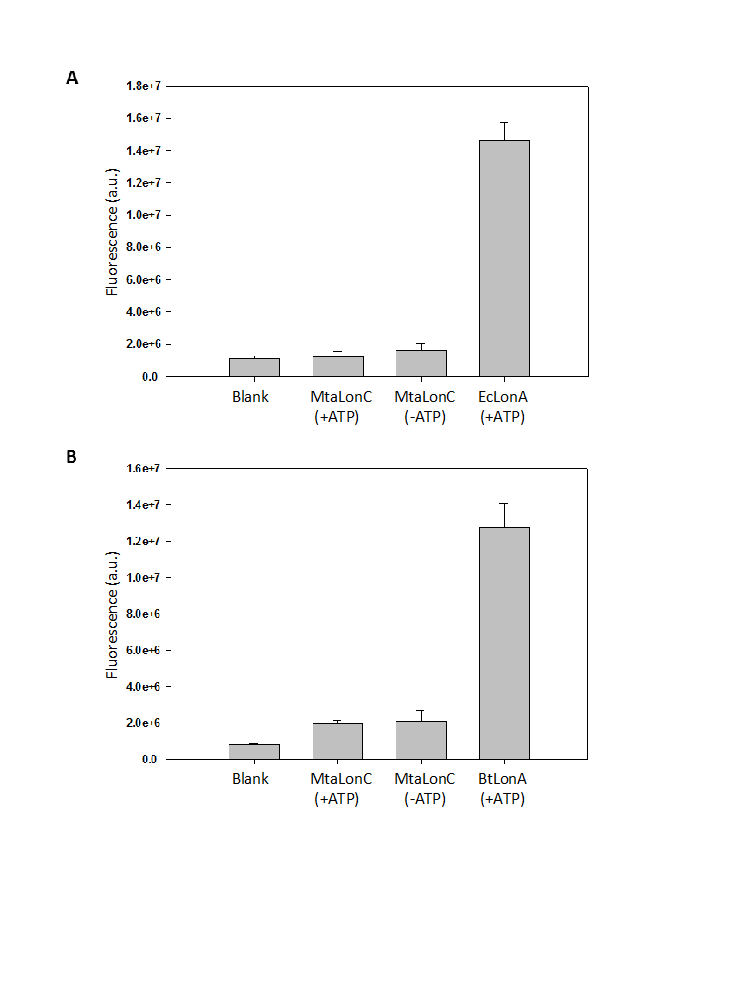

Supplement: Figure S5 — MtaLonC cannot degrade dephosphorylated casein. Protease activity of MtaLonC (50 µg) against dephosphorylated FITC-casein was assayed in the absence or presence of ATP. The fluorescence was measured as described previously (J Biol Chem 279 (2004): 34903–34912; Eur J Biochem 271 (2004): 834–844). Emission was recorded at 525 nm. (A) Cleavage of dephosphorylated FITC-casein at 37°C. EcLonA (4 µg) was used as a positive control. (B) Degradation of the substrate at 55°C. BtLonA (4 µg) was used as a positive control. Blank, no added enzyme. (TIF) [file pone.0040226.s005.tif]

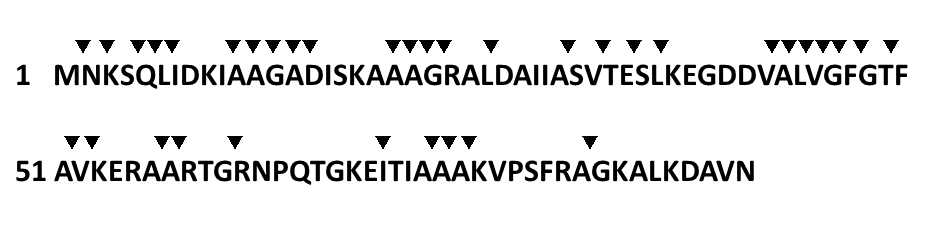

Supplement: Figure S6 — The cutting site of MtaLonC in E. coli Huβ. E. coli Huβ were degraded by MtaLonC in the buffer containing 50 mM Tris HCl, 10 mM Na2HPO4, pH 8.0 for 17 hours and analyzed by mass spectrometry. (TIF) [file pone.0040226.s006.tif]

**Table S1.**


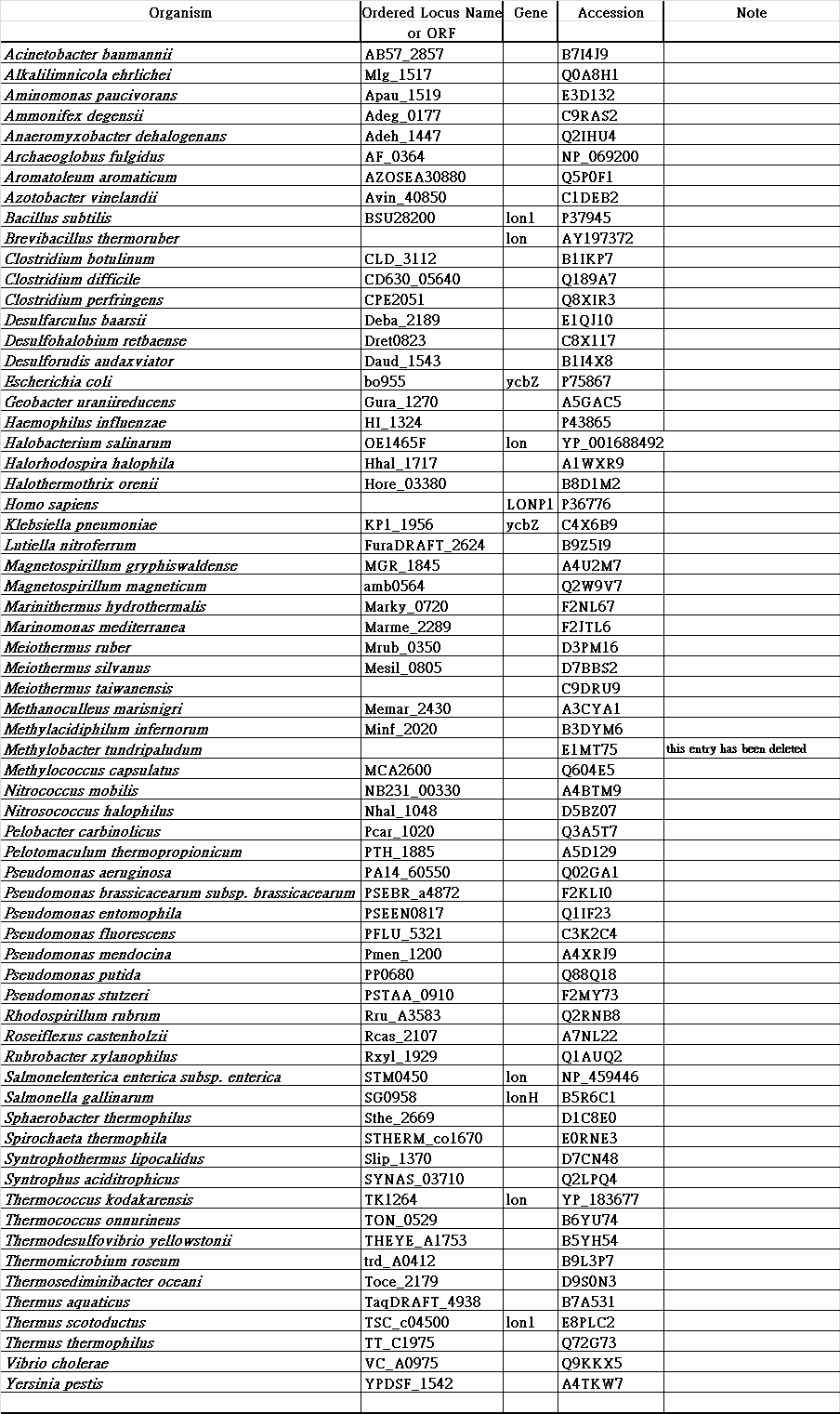

Supplement: Table S1 — The accession codes for the genes in phylogenetic tree. (DOCX) [file pone.0040226.s007.docx]
